# Supplementary material for: Vibrational hierarchy leads to dual-phonon transport in low thermal conductivity crystals
Source: Nat Commun. 2020 May 22;11:2554. doi: 10.1038/s41467-020-16371-w (PMC7244571; doi:10.1038/s41467-020-16371-w)
Supplement: Supplementary file 1 — Supplementary Information [file 41467_2020_16371_MOESM1_ESM.pdf]

Supplementary Information

Vibrational hierarchy leads to dual-phonon transport in low thermal conductivity crystals

Luo *et al*.

Supplementary Notes

Supplementary Note 1: Interests and challenges in κ_L-prediction of $La_2Zr_2O_7$

In this study, $La_2Zr_2O_7$, a state-of-the-art thermal barrier coating (TBC) material for the gas turbine technology, is chosen to demonstrate our proposed dual-phonon theory. $La_2Zr_2O_7$ is a typical complex-structure oxide ceramic with low κ_L (~1 to 2 $W\cdot m^{-1}\cdot K^{-1}$ at high-temperature, $T > 1000$ K)[1-3]. Plus, it has high melting point, high sintering resistance, good phase stability, and promising ability to inhibit infiltration of calcium-magnesium aluminosilicate (CMAS), promoting its application as TBC.[4,5] So far, κ_L calculations based on empirical models, such as Cahill's model,[6] Clarke's model,[7] Slack's model[8], and Callaway's model[9], have been performed.[10-13] Nevertheless, there hasn't been a physics-based explanation for the experimentally observed flattening-out of κ_L~T dependence.[1-3] Besides, the contribution from optical phonons was neglected in these studies, which might result in inaccurate κ_L prediction, given that the optical phonons might provide essential scattering channels for acoustic phonons, and carry a lot of heat in materials where the phonon conductivity is suppressed.[14-17] Meanwhile, the anharmonicity (quantified by the Grüneisen constant) of lattice vibrations of $La_2Zr_2O_7$ are reported to vary by several times for different vibrational modes,[13] which is expected to yield pronounced modal-level vibrational hierarchy. Therefore, a mode-by-mode investigation on the physics of hierarchical vibrations as well as their thermal transport mechanisms in this material attracts the interest from both theoretical and engineering sides.

Supplementary Note 2: $D_{\mathrm{Diff}}^{\mathrm{AF}}$ and $\kappa_{\mathrm{L}}^{\mathrm{AF}}$ calculated from Allen-Feldman theory

The per-mode thermal diffusivity ($D_{\mathrm{Diff}}^{\mathrm{AF}}$) of $La_2Zr_2O_7$ calculated using the Allen-Feldman theory initially exhibits a $1/\nu^{-n}$ (parameter n is estimated to be 2 or 4) trend at the low-frequency range, which corresponds to the propagating behavior; beyond that it does not show clear frequency dependence (see Supplementary Figure 1). This is a typical scaling behavior seen in many materials with certain degree of disorder.[18,19] Based on these results, κ_L could be directly calculated from $\kappa_{\mathrm{L}}^{\mathrm{AF}} = \sum_{j=1}^{3N} C_{\mathrm{s}}(j) D_{\mathrm{Diff}}^{\mathrm{AF}}(j)$,

where $C_s(j)$ is the per-mode specific heat (see Equation (4) in the main text), and j sums over all the vibrational modes (N denotes the number of atoms in the simulated unit cell). Results of calculated κ_L^{AF} are shown in the inset of Supplementary Figure 1. This could be seen as the amorphous limit for solids. Herein, the ab-initio derived thermal diffusivity is temperature independent, and thus the trend of κ_L^{AF} is dominated by temperature dependence of specific heat.

Supplementary Note 3: The dual-phonon theory for Tl_3VSe_4

Our proposed dual-phonon theory is also validated on Tl_3VSe_4, a potential thermoelectric material with ultra-low κ_L. It has also been used as the model material in Mukhopadhyay *et al.*'s work,[20] to develop the two-channel model. The calculated phonon dispersions and density of states for Tl_3VSe_4 are presented in Supplementary Figure 4, where the calculated phonon frequencies at the Γ point show good agreement with the experimental data from Raman spectroscopy measurements[20]. Large amounts of phonon modes have very small mean free path (l) or thermal diffusivity (D_{Phon}), which could be distinguished as diffuson-like phonon as opposed to normal phonons. At T=300 K, the number of these "ill-defined-in-space" modes account for 74.06%, 44.74% and 66.90% of the total number of modes, respectively, based on the three criteria we proposed (I. l-λ criterion, II. l-a_{min} criterion, and III. D_{Phon}-D_{Diff} criterion coupled with the random-walk theory). The fractions rise up to 80.48%, 57.07% and 74.02% at T=500 K. The results are presented in Supplementary Figure 5, together with the hierarchical features of the phonon group velocity (v_g) and phonon relaxation time (τ) for each mode.

κ_L of Tl_3VSe_4 is calculated using our proposed dual-phonon theory, by incorporating D_{Diff} data computed from the random-walk theory. The calculated κ_L shows a milder-than-T^{-1} temperature dependence, in good agreement with the experimental data[20] (see Supplementary Figure 6 and Supplementary Table 1), as compared with the serious underestimation of κ_L and erroneous κ_L~T dependence predicted from phonon Boltzmann transport equation (BTE) theory. As shown in Supplementary Figure 6(b), the normal phonons are responsible for heat transfer at

around T<300 K, beyond which the diffuson-like phonons start to take over. We also calculate κ_L according to the two-channel model[20]. In this model, heat conduction from the hopping channel (κ_{hop}) are calculated either from Cahill's formula, which requires v_g of acoustic phonons as input; or from Einstein's formula, which requires defining the Einstein temperature (θ_E), both of which are rooted in the random-walk theory. Herein for Tl_3VSe_4, v_g for the one longitudinal acoustic phonon and two transverse acoustic phonons are extracted from the calculated phonon dispersions, giving 2169 $m \cdot s^{-1}$, 945 $m \cdot s^{-1}$ and 1245 $m \cdot s^{-1}$, respectively; and θ_E is estimated from the calculated low-temperature specific heat (Supplementary Figure 7), yielding θ_E=34 K, all in agreement with previous reports[20]. κ_{hop} calculated using these two sets of input data are plotted as the upper and lower bound of κ_L, respectively, for the two-channel model, shown in the green shaded area in Supplementary Figure 6(a). For comparison, results directly extracted from Mukhopadhyay *et al.*'s work[20] are also presented as yellow shaded area, which agree with our values. One thing to notice is that in our study, phonon-phonon interactions and phonon scattering from natural isotope variations are considered; whereas in Mukhopadhyay *et al.*'s work[20], phonon scatterings from boundaries are also included, which is expected to suppress the κ_L at lower temperatures. This could explain the difference between our and the referenced results at lower temperature range.

Supplementary Note 4: Structural origin of the small-l or small-D_{Phon} vibrations

To further link the diffuson-like phonons in $La_2Zr_2O_7$ with its crystal structure, we perform a detailed analysis on the phonon dispersion and scattering processes. As shown in Supplementary Figure 2, the complex crystal structure (22 atoms in a primitive unit cell) of $La_2Zr_2O_7$ yields a large number of flattened vibration branches with low v_g, especially for high-frequency phonons. Plus, a number of low-frequency optical phonons overlap with acoustic phonons. A previous study has shown that these low-frequency phonons are strongly anharmonic, and could be primarily attributed to vibrations of La^{3+} ions in particularly loose bonding environment,[13] as compared to other relatively strong interatomic bonds.

Supplementary Figure 8 shows mode-resolved phonon scattering rate ($1/\tau$) of

$La_2Zr_2O_7$. Here, the high-frequency optical phonons overall exhibit higher scattering rates than the low-frequency acoustic and optical phonons (v<4 THz). For instance, the $1/\tau$ values for phonons of v>15 THz fall in the range of 1 to 10 ps^{-1} at T=300 K; whereas those for phonons of v<4 THz fall in the range of 10^{-3} to 1 ps^{-1}. Results for T=1500 K show a similar trend. This could be understood from the higher chance of high-frequency phonons to satisfy the conservation rule of energy and momentum, and thus participate extensively in the scattering processes of $La_2Zr_2O_7$. Of the most interest, there are obvious bumps of $1/\tau$ values at phonon frequencies of v~2 THz and v~5 THz, indicative of abruptly increased phonon scattering among low-frequency acoustic and optical phonons. A reflection on phonon dispersion curves reveals that the former corresponds with the avoided crossing between low-frequency optical phonons with TA phonons, causing TA dispersion curves to flatten out as they move away from the BZ center; while the latter could be attributed to the severe bunching of low-frequency optical phonons, leading to intense phonon scattering. Furthermore, the total phonon scattering rate at T=300 K is decomposed into absorption and emission processes, and the results are plotted in the inset of Supplementary Figure 8. As shown, the low-frequency acoustic and optical phonons (v<4 THz) are mainly involved in the absorption process, being scattered into phonons with higher frequencies when combining with another phonon. In comparison, the bunched low-frequency optical phonons (v~5 THz) are mainly involved in the emission process, being scattered into two phonons with lower frequencies. These findings suggest that for $La_2Zr_2O_7$, the vibrational modes across wide frequency ranges undergo severe scattering, leading to high scattering rates and small τ. Plus, the role of the low-frequency optical phonons is accentuated as they provide important scattering channels for acoustic phonons.

Supplementary Note 5: Implications for prediction and design of low-κ_L TBC materials

Currently, prediction of κ_L for TBC materials frequently incorporates first-principles calculations coupled with empirical models. As most widely used, Clarke's model[7] proposed the concept of minimum thermal conductivity (κ_{min}), by replacing

different atoms in a molecule with one equivalent atom, and assuming that phonon mean free path approaches the average interatomic spacing, and the per-atom specific heat approaches $3k_B$ at high temperatures. This model gives the lowest-bound of κ_L as a constant for crystalline, in reasonable agreement with experimental data at high-temperature limit; and provides technologically important guidelines for low-κ_L materials, based on both theoretically and experimentally accessible materials properties, i.e. material density, average atomic mass and Young's modulus. Meanwhile, simulations of the temperature-dependent κ_L are enabled by Slack's model[8], which assumes that Umklapp scatterings (with relaxation time τ_U scaling with T^{-1}) among acoustic phonons dominate heat transport above the Debye temperature, or by Debye-Callaway model[9], whose formula enables distinguishing the contribution from each acoustic branch. These models neglect the direct participation of optical phonons due to their low group velocity. Based on these models, TBC materials has typically been predicted to have a $\kappa_L \propto T^{-1}$ trend with increasing temperature, then bounded by κ_{min} once reached. Such methodologies are helpful in the rudimental material screening, yet they bare several deficiencies as noted in Supplementary Note 1.

Our proposed dual-phonon theory improves the methodology of κ_L-prediction for TBC materials in the following aspects: (1) the scatterings of both acoustic and optical modes are considered; (2) more realistic $\kappa_L \sim T$ relationship could be simulated by considering the hierarchy of lattice vibrations, i.e. normal phonons treated in the BTE theory versus diffuson-like phonons treated in the diffusion theory for thermal transport; (3) the physical insight of thermal conduction could be obtained from modal vibrational properties, i.e. scattering channels, relaxation time, *etc*..

Supplementary Note 6: Computational details for Tl_3VSe_4

For density functional theory calculations of Tl_3VSe_4, we employ the projector augmented wave (PAW) method[21] within the Perdew, Burke and Ernzerhof (PBE)[22] form of generalized gradient approximation (GGA) as implemented in VASP[23]. For structural optimization, the energy cutoff is set as 500 eV, the convergence thresholds are 10^{-10} eV for total energy and 10^{-5} eV/Å for maximum ionic Hellmann-Feynman

forces, and Monkhorst Pack grids of 11 × 11 × 11 is used to sample the Brillouin zone (BZ). The lattice parameter of Tl_3VSe_4 (space group $I\bar{4}3m$) is relaxed to be a=7.891 Å, in agreement with reported results (a=7.904 Å)[20]. The harmonic and anharmonic interatomic force constants (IFCs) are calculated via the finite displacement method using VASP-phonopy[24] and VASP-thirdorder [25] interfaces, respectively, based on 2 × 2 × 2 supercells and 5 × 5 × 5 Monkhorst Pack grids. Specially for anharmonic IFCs, interatomic interactions up to the seventh nearest neighbors (7th NN) are taken into account, corresponding to a cutoff radius (r^{cutoff}) of 7.36 Å. κ_L are calculated under the relaxation time approximation (RTA), which typically works well for low κ_L materials. Scattering from isotopes and the non-analytical corrections are incorporated. Here we note that the computational settings for Tl_3VSe_4 in this study are comparable with Mukhopadhyay *et al.*'s work.[20]

Supplementary Note 7: Convergence test for the Lorentzian broadening factor

We did a convergence test on the Lorentzian broadening factor using a range from $1.1\Delta_{avg}$ to $4.4\Delta_{avg}$ for some wave vectors. The Supplementary Figure 9 shows the calculated frequency-dependent D_{Diff}^{AF} of $La_2Zr_2O_7$ with Lorentzian broadening factors of $2.2\Delta_{avg}$, $3.3\Delta_{avg}$, and $4.4\Delta_{avg}$, for the modes at **q**=(0.25, 0.25, 0.25) and **q**=(0.5, 0.5, 0.5). We observe reasonable convergence. Moreover, the uncertainties of the calculated κ_L^{AF} are less than 6.2% for **q**=(0.25, 0.25, 0.25) and 5.6% for **q**=(0.5, 0.5, 0.5), respectively. Note that the data for $1.1\Delta_{avg}$ have not converged yet, and thus is not shown in the figure. Hence, we use a broadening factor of $3.3\Delta_{avg}$ in our calculations. We also note that it has been acknowledged that, a frequency-dependent Lorentzian broadening width may be useful[18,26].

Supplementary Figures

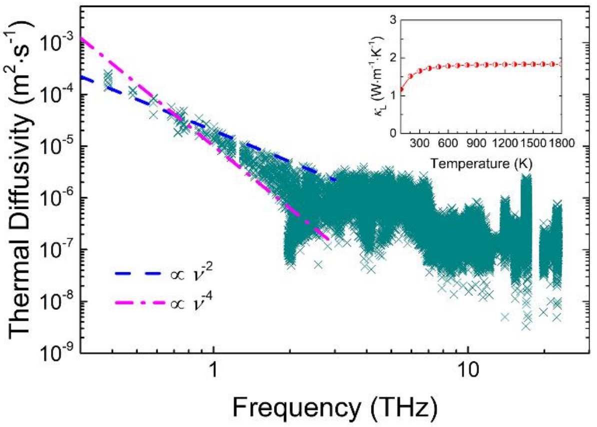

Supplementary Figure 1. Allen-Feldman theory on $La_2Zr_2O_7$. The thermal diffusivity (D_{Diff}) for each vibrational mode is calculated from the Allen-Feldman formula. Also shown are extrapolations based on an ν^{-2} and ν^{-4} scaling (ν denotes vibrational frequency). Inset: Temperature-dependent thermal conductivity (κ_{L}) calculated directly from the Allen-Feldman theory. Source data are provided as a Source Data file.

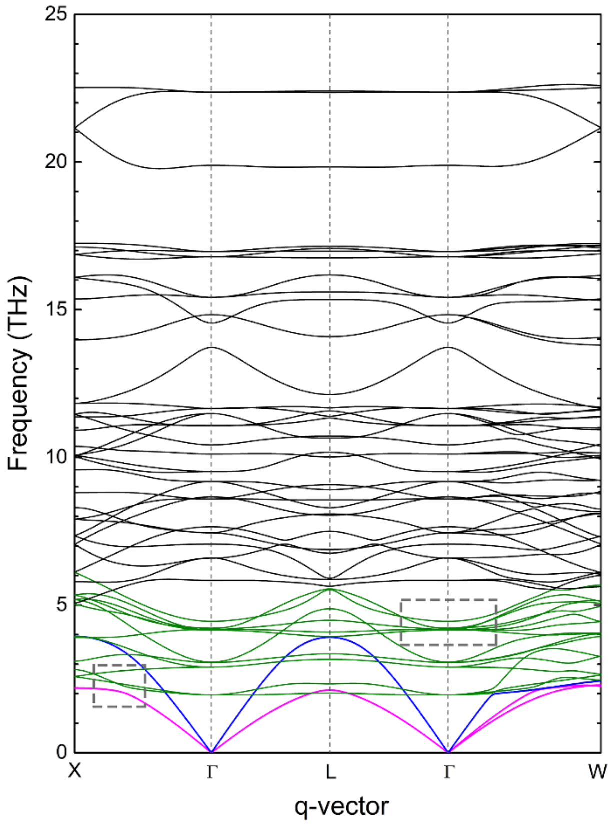

Supplementary Figure 2. Phonon dispersion of $La_2Zr_2O_7$. The calculated phonon dispersion of $La_2Zr_2O_7$ along the high-symmetry directions in the Brillouin zones (BZ), with the LO-TO (LO denotes longitudinal optical mode; TO denotes transverse acoustic mode) splitting effects considered using non-analytical corrections. The transverse acoustic (TA), longitudinal acoustic (LA), low-frequency optical and high-frequency optical phonons are highlighted in magenta, blue, green and black lines, respectively. The dashed grey rectangles exemplify the avoided crossing between the low-frequency optical and acoustic phonons, as well as the bunching of the low-frequency phonons. The group velocities for acoustic phonons could be calculated from the slope of the acoustic phonon dispersion around Γ point, averaged along each BZ direction, giving TA: 3837 $m \cdot s^{-1}$ and 3969 $m \cdot s^{-1}$; and LA: 6872 $m \cdot s^{-1}$. Source data are provided as a Source Data file.

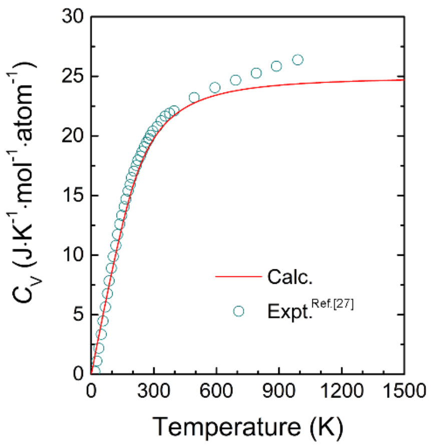

Supplementary Figure 3. Heat capacity of $La_2Zr_2O_7$. The calculated heat capacity (C_V) of $La_2Zr_2O_7$ as a function of temperature, in comparison with the experimental data[27]. Source data are provided as a Source Data file.

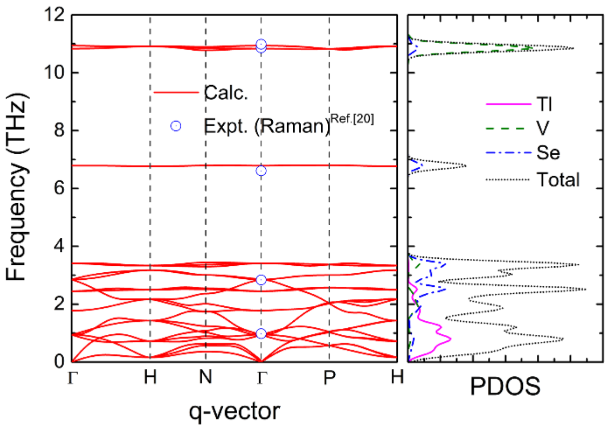

Supplementary Figure 4. Phonon dispersion of Tl_3VSe_4. The calculated phonon dispersion for Tl_3VSe_4 along the high-symmetry directions in the Brillouin zones (BZ), with the LO-TO splitting effects considered using non-analytical corrections; and the corresponding phonon density of states projected onto each atom. The phonon frequencies from Raman spectroscopy measurements[20] are marked by blue circles. Source data are provided as a Source Data file.

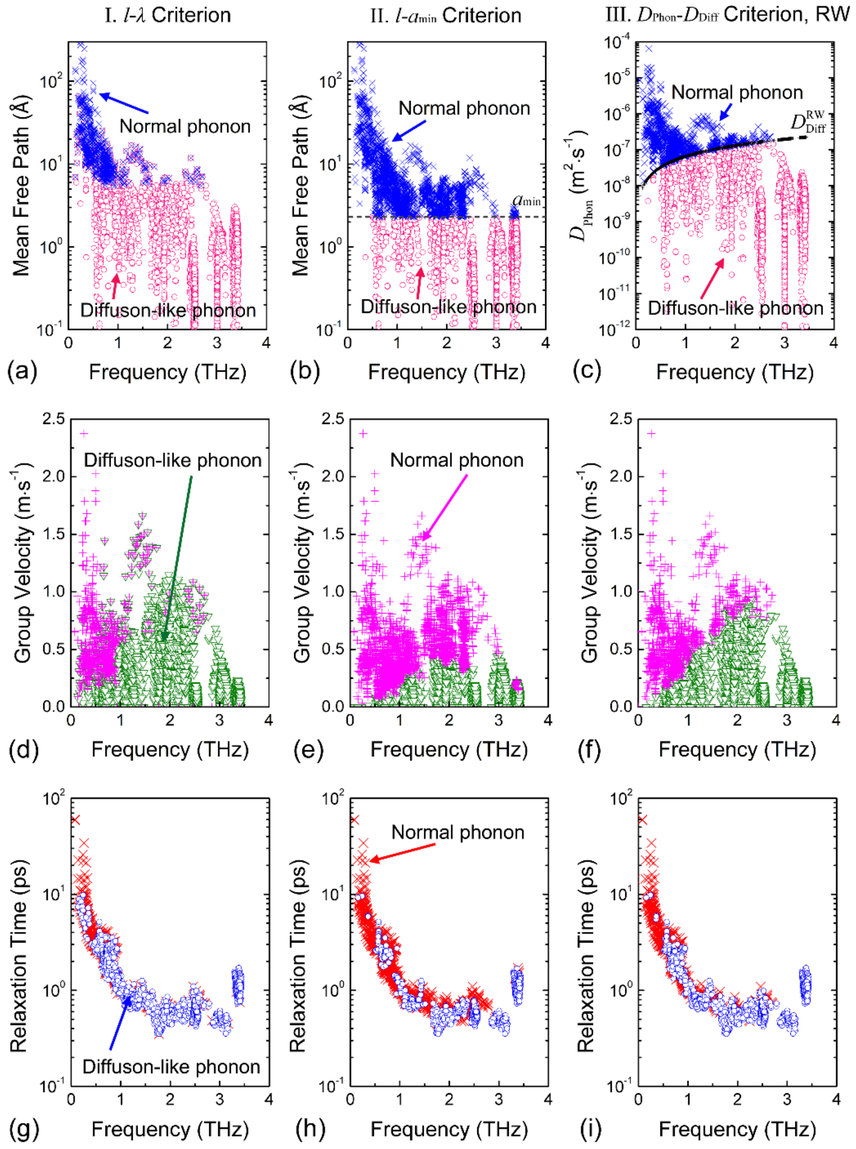

Supplementary Figure 5. Hierarchy of lattice vibrations for Tl_3VSe_4. (a) (b) (c) The calculated phonon mean free path (l) and thermal diffusivity (D_{Phon}) for Tl_3VSe_4 at T=300 K, to distinguish between the normal phonons versus diffuson-like phonons according to our proposed criterion I, II, and III, respectively. Herein, λ denotes the vibrational wavelength; and the diffuson thermal diffusivity (D_{Diff}) is calculated from the random-walk (RW) theory. The minimum interatomic spacing (a_{min}=2.3 Å) of Tl_3VSe_4 is drawn in a dashed line as a guide for the eye. (d) (e) (f) The calculated phonon group velocity (v_g) and (g) (h) (i) relaxation time (τ) at T=300 K for each vibrational mode of Tl_3VSe_4, based on criterion I, II, and III, respectively. Source data are provided as a Source Data file.

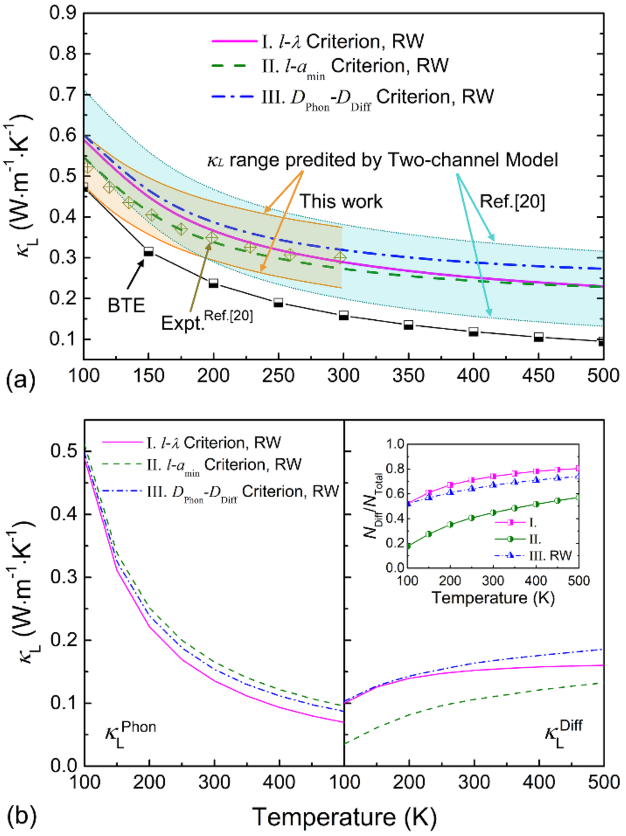

Supplementary Figure 6. Lattice Thermal conductivity of Tl_3VSe_4. (a) The calculated temperature-dependent lattice thermal conductivity (κ_L) for Tl_3VSe_4 using our proposed dual-phonon theory coupled with the random-walk (RW) treatment of diffuson-like phonons, in comparison with the results from the BTE theory, the two-channel model, and experimental data[20]. The green shaded area outlines the range of κ_L predicted by the two-channel mode using our calculated data for Tl_3VSe_4 as inputs, whereas the yellow shaded area presents the results extracted from Mukhopadhyay *et al.*'s work[20] for comparison. (b) The contribution to the total κ_L from normal phonons (κ_L^{Phon}) and diffuson-like phonons (κ_L^{Diff}). Inset: The number of diffuson-like phonons (N_{Diff}) divided by the number of all vibrational modes ($N_{\mathrm{Total}}=N_{\mathrm{Phon}}+N_{\mathrm{Diff}}$) for Tl_3VSe_4, calculated as a function of the temperature. Source data are provided as a Source Data file.

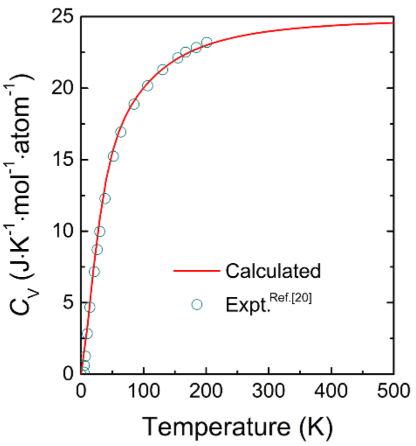

Supplementary Figure 7. Heat capacity of Tl_3VSe_4. The calculated heat capacity (C_V) of Tl_3VSe_4 as a function of the temperature, which agrees well with the experimental data[20]. Source data are provided as a Source Data file.

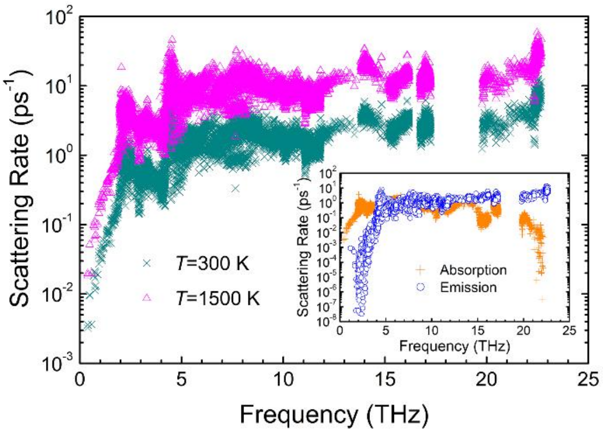

Supplementary Figure 8. Scattering rate of $La_2Zr_2O_7$. The calculated vibrational scattering rates for $La_2Zr_2O_7$, and (inset) classified into absorption and emission processes for T=300 K. Source data are provided as a Source Data file.

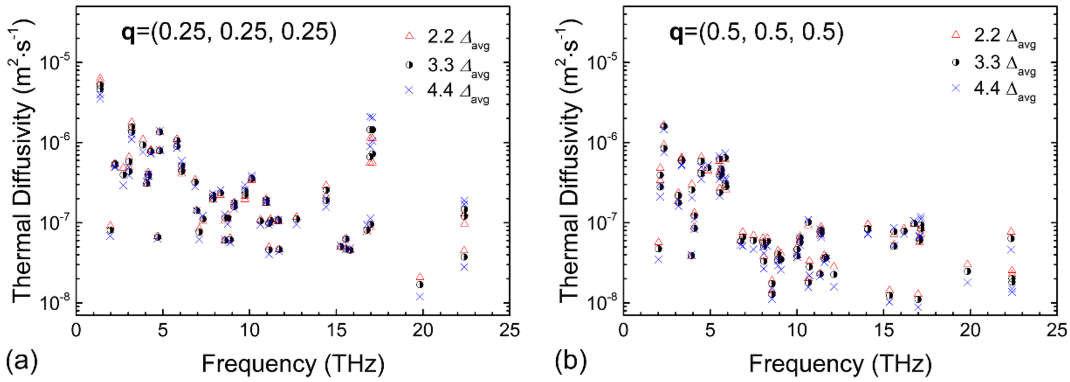

Supplementary Figure 9. Convergence test with respect to the Lorentzian broadening width. The calculated frequency-dependent $D_{\mathrm{Diff}}^{\mathrm{AF}}$ of $La_2Zr_2O_7$ with Lorentzian broadening factors of $2.2\Delta_{\mathrm{avg}}$, $3.3\Delta_{\mathrm{avg}}$ and $4.4\Delta_{\mathrm{avg}}$, for the modes at (a) **q**=(0.25, 0.25, 0.25) and (b) **q**=(0.5, 0.5, 0.5). Herein, Δ_{avg} denotes the average mode frequency interval. Source data are provided as a Source Data file.

Supplementary Tables

Supplementary Table 1. The calculated κ_L of Tl_3VSe_4 (T=300 K) using our proposed dual-phonon theory coupled with the random-walk (RW) treatment of diffuson-like phonons, and the parameter n in the $\kappa_L \sim T^{-n}$ dependence is fitted within T=100~300 K. The results calculated from the phonon BTE theory and the experimental data[20] are presented for comparison.

Method		κ_L ($W \cdot m^{-1} \cdot K^{-1}$) T=300 K	n ($\kappa_L \sim T^{-n}$) T=100~300 K
Dual-phonon theory	I. l-λ Criterion, RW	0.288	0.672
	II. l-a_{min} Criterion, RW	0.272	0.662
	III. D_{Phon}- D_{Diff} Criterion, RW	0.318	0.605
BTE		0.158	0.997
Expt.[20]		0.298	0.560

Supplementary References

[1] C. Wan, W. Zhang, Y. Wang, Z. Qu, A. Du, R. Wu, and W. Pan, Glass-like thermal conductivity in ytterbium-doped lanthanum zirconate pyrochlore, Acta Mater. **58**, 6166-6172 (2010)

[2] H. Lehmann, D. Pitzer, G. Pracht, R. Vassen, and D. Stöver, Thermal conductivity and thermal expansion coefficients of the lanthanum rare-earth-element zirconate system, J. Am. Ceram. Soc. **86**, 1338-1344 (2003)

[3] G. Suresh, G. Seenivasan, M. V. Krishnaiah, and P. S. Murti, Investigation of the thermal conductivity of selected compounds of gadolinium and lanthanum, J. Nucl. Mater **249**, 259-261 (1997)

[4] N. P. Bansal and D. Zhu, Effects of doping on thermal conductivity of pyrochlore oxides for advanced thermal barrier coatings, Mater. Sci. Eng. A **459**, 192-195 (2007)

[5] S. Kramer, J. Yang, and C. G. Levi, Infiltration-inhibiting reaction of gadolinium zirconate thermal barrier coatings with CMAS melts, J. Am. Ceram. Soc. **91**, 576-583 (2008)

[6] D. G. Cahill, S. K. Watson, and R. O. Pohl, Lower limit to the thermal conductivity of disordered crystals, Phys. Rev. B **46**, 6131-6140 (1992)

[7] D. R. Clarke, Materials selection guidelines for low thermal conductivity thermal barrier coatings, Surf. Coat. Tech. **163**, 67-74 (2003)

[8] G. A. Slack, Nonmetallic crystals with high thermal conductivity, J. Phys. Chem. Solids **34**, 321-335 (1973)

[9] D. T. Morelli, J. P. Heremans, and G. A. Slack, Estimation of the isotope effect on the lattice thermal conductivity of group IV and group III-V semiconductors, Phys. Rev. B **66**, 195304 (2002)

[10] B. Liu, J. Y. Wang, Y. C. Zhou, T. Liao, and F. Z. Li, Theoretical elastic stiffness, structure stability and thermal conductivity of $La_2Zr_2O_7$ pyrochlore, Acta Mater. **55**, 2949-2957 (2007)

[11] J. Feng, B. Xiao, C. Wan, Z. Qu, Z. Huang, J. Chen, R. Zhou, and W. Pan, Electronic structure, mechanical properties and thermal conductivity of $Ln_2Zr_2O_7$

(Ln=La, Pr, Nd, Sm, Eu and Gd) pyrochlore, Acta Mater. **59**, 1742-1760 (2011)

[12] J. Feng, B. Xiao, R. Zhou, and W. Pan, Thermal conductivity of rare earth zirconate pyrochlore from first principles, Scripta Mater. **68**, 727-730 (2013)

[13] G. Lan, B. Ouyang, and J. Song, The role of low-lying optical phonons in lattice thermal conductance of rare-earth pyrochlores: a first-principle study, Acta Mater. **91**, 304-317 (2015)

[14] L. Lindsay, D. A. Broido, and T. L. Reinecke, Ab initio thermal transport in compound semiconductors, Phys. Rev. B **87**, 165201 (2013)

[15] W. Li, L. Lindsay, D. A. Broido, D. A. Stewart, and N. Mingo, Thermal conductivity of bulk and nanowire $Mg_2Si_xSn_{1-x}$ alloys from first principles, Phys. Rev. B **86**, 174307 (2012)

[16] T. Shiga, J. Shiomi, J. Ma, O. Delaire, T. Radzynski, A. Lusakowski, K. Esfarjani, and G. Chen, Microscopic mechanism of low thermal conductivity in lead telluride, Phys. Rev. B **85**, 155203 (2012)

[17] L. Feng, T. Shiga, and J. Shiomi, Phonon transport in perovskite $SrTiO_3$ from first principles, Appl. Phys. Express **8**, 071501 (2015)

[18] J. M. Larkin and A. J. H. McGaughey, Thermal conductivity accumulation in amorphous silica and amorphous silicon, Phys. Rev. B **89**, 144303 (2014)

[19] T. Zhu and E. Ertekin, Mixed phononic and non-phononic transport in hybrid lead halide perovskites: glass-crystal duality, dynamical disorder, and anharmonicity, Energy Environ. Sci. **12**, 216-229 (2019)

[20] S. Mukhopadhyay, D. S. Parker, B. C. Sales, A. A. Puretzky, M. A. McGuire, and L. Lindsay, Two-channel model for ultralow thermal conductivity of crystalline Tl_3VSe_4, Science **360**, 1455-1458 (2018)

[21] P. E. Blöchl, Projector augmented-wave method, Phys. Rev. B **50**, 17953-17979 (1994)

[22] J. P. Perdew, K. Burke, and M. Ernzerhof, Generalized gradient approximation made simple. Phys. Rev. Lett. **77**, 3865-3868 (1996)

[23] G. Kresse and J. Furthmüller, Efficient iterative schemes for ab initio total-energy calculations using a plane-wave basis set, Phys. Rev. B **54**, 11169-11186 (1996)

[24] A. Togo and I. Tanaka, First principles phonon calculations in materials science, Scripta Mater. **108**, 1-5 (2015)

[25] W. Li, J. Carrete, N. A. Katcho, and N. Mingo, ShengBTE: A solver of the Boltzmann transport equation for phonons, Comput. Phys. Commun. **185**, 1747-1758 (2014)

[26] M. Simoncelli, N. Marzari, and F. Mauri, Unified theory of thermal transport in crystals and glasses, Nat. Phys. **15**, 809-813 (2019)

[27] S. Nandi, Y. M. Jana, and H. C. Gupta, Lattice dynamical investigation of the Raman and infrared wave numbers and heat capacity properties of the pyrochlores $R_2Zr_2O_7$ (R=La, Nd, Sm, Eu), J. Phys. Chem. Solids **115**, 347-354 (2018)
